# Phosphatidylinositol-4,5-bisphosphate is required for KCNQ1/KCNE1 channel function but not anterograde trafficking

**DOI:** 10.1371/journal.pone.0186293

**Published:** 2017-10-11

**Authors:** Alice A. Royal, Andrew Tinker, Stephen C. Harmer

**Affiliations:** William Harvey Research Institute, The Heart Centre, Barts and the London School of Medicine and Dentistry, Queen Mary University of London, London, United Kingdom; Indiana University School of Medicine, UNITED STATES

## Abstract

The slow delayed-rectifier potassium current (*I*_Ks_) is crucial for human cardiac action potential repolarization. The formation of *I*_Ks_ requires co-assembly of the KCNQ1 α-subunit and KCNE1 β-subunit, and mutations in either of these subunits can lead to hereditary long QT syndrome types 1 and 5, respectively. It is widely recognised that the KCNQ1/KCNE1 (Q1/E1) channel requires phosphatidylinositol-4,5-bisphosphate (PIP_2_) binding for function. We previously identified a cluster of basic residues in the proximal C-terminus of KCNQ1 that form a PIP_2_/phosphoinositide binding site. Upon charge neutralisation of these residues we found that the channel became more retained in the endoplasmic reticulum, which raised the possibility that channel–phosphoinositide interactions could play a role in channel trafficking. To explore this further we used a chemically induced dimerization (CID) system to selectively deplete PIP_2_ and/or phosphatidylinositol-4-phosphate (PI(4)P) at the plasma membrane (PM) or Golgi, and we subsequently monitored the effects on both channel trafficking and function. The depletion of PIP_2_ and/or PI(4)P at either the PM or Golgi did not alter channel cell-surface expression levels. However, channel function was extremely sensitive to the depletion of PIP_2_ at the PM, which is in contrast to the response of other cardiac potassium channels tested (Kir2.1 and Kv11.1). Surprisingly, when using the CID system *I*_Ks_ was dramatically reduced even before dimerization was induced, highlighting limitations regarding the utility of this system when studying processes highly sensitive to PIP_2_ depletion. In conclusion, we identify that the Q1/E1 channel does not require PIP_2_ or PI(4)P for anterograde trafficking, but is heavily reliant on PIP_2_ for channel function once at the PM.

## Introduction

Cardiac repolarisation is determined by a number of K^+^ currents, including the slow delayed-rectifier potassium current (*I*_Ks_), the rapid delayed-rectifier potassium current (*I*_Kr_) and the inward-rectifier potassium current (*I*_K1_). The channel underlying *I*_Ks_ is a heteromultimeric complex comprised of a canonical voltage-gated K^+^ channel α-subunit, KCNQ1 (Q1) (Kv7.1), and a β-subunit, KCNE1 (E1) [1, 2]. In a related vein, the Kv11.1 α-subunit forms the human ether-a-go-go-related gene (hERG) channel and constitutes the *I*_Kr_ current in human ventricular myocytes [3]. *I*_K1_ is a classic inward rectifier K^+^ current and is likely constituted predominantly by Kir2.1 [4].

*I*_Ks_ is so named because of its slow kinetics, which allow the current to accumulate at high heart rates. Notably, *I*_Ks_ is augmented through increased adrenergic drive [5–7]. As such, *I*_Ks_ is thought to be particularly important during exercise, when it plays a major role in action potential and QT interval shortening [8]. This is supported by the fact that most cases of sudden death in patients with long QT syndrome type 1 (LQT1; caused by mutations in Q1) occur during exercise [9].

Numerous ion channels are regulated by phosphatidylinositols [10–12]. Phosphatidylinositols are a family of acidic phospholipids, each member differing in the phosphorylation of its *myo*-inositol ring. These phospholipids are present on the cytosolic face of cellular membranes and are involved in many biological processes, including vesicle formation and trafficking [13]. Phosphatidylinositol (PI) is synthesised in the endoplasmic reticulum (ER) and is subsequently transported via specific transfer proteins or vesicles. PI kinases then phosphorylate PI to generate different PI species in the relevant cellular location. For example, phosphatidylinositol-4,5-bisphosphate (PIP_2_ or PI(4,5)P_2_) is predominantly present in the plasma membrane (PM), whilst phosphatidylinositol-4-phosphate (PI(4)P) is present in the PM but is also enriched at the Golgi [13, 14].

It is well established that the Q1/E1 channel requires the presence of PIP_2_ to function, and it is thought that it acts to stabilise the channel in an open state [15–17]. Other KCNQ channels also require PIP_2_ for voltage-dependent activation, and PIP_2_ depletion is likely the main mechanism by which the muscarinic acetylcholine receptor (M_1_) induces M-channel (KCNQ2/3) closure [15, 18]. In our earlier work we defined a PIP_2_-binding region in the proximal C-terminus of the channel, with the most important residues in this region being Lys358 and Arg360 [19]. Even though PIP_2_ bound to the channel with the highest affinity, other phosphoinositide species could also bind [19]. Furthermore, we showed that the homologous residues in KCNQ2/3 are also likely to form a phosphoinositide binding site [20, 21]. In a recent structural study of KCNQ1 using cryo-electron microscopy, the voltage sensor domains were uncoupled from the closed pore region in the absence of PIP_2_ [22]. Therefore, it is clear that PIP_2_, through direct binding to a KCNQ potassium channel domain, can modulate channel opening [23, 24]. What is less clear, however, is whether binding of PIP_2_ in the PM and/or PI(4)P in the Golgi plays a role in the anterograde trafficking of KCNQ channels. This is a relevant issue for KCNQ1 as a number of LQT1-causing mutations reside in PIP_2_ binding sites [25], and we have shown that the impaired delivery of mutant channels to the PM and their retention in the ER is an important disease mechanism [26, 27].

The established methods for assessing the function of phosphoinositides, such as activation of a G_q/11_-coupled G-protein coupled receptor (GPCR) or the addition of wortmannin, tend to be indirect and lack specificity. For example, the kinase inhibitory actions of wortmannin are wider ranging than previously thought [28] and the phospholipase C (PLC)-mediated depletion of PIP_2_ upon activation of the M_1_ receptor (a G_q/11_-GPCR) additionally produces diacylglycerol, activates protein kinase C and mobilises Ca^2+^ from intracellular stores. The identification of the voltage-sensitive phosphatase (VSP) from *Ciona intestinalis* (Ci-VSP) [29] has provided a tool that can deplete PIP_2_ in a more specific manner. Ci-VSP localises to the PM and can rapidly deplete PIP_2_ and phosphatidylinositol-3,4,5-trisphosphate (PI(3,4,5)P_3_) upon membrane depolarization [30]. VSPs, in conjunction with patch clamp recording, have been used to determine the PIP_2_ dependence of a range of ion channels (for example [31] and [32]). However, despite the obvious advantages of VSPs their PM localisation and requirement for membrane depolarisation can be limiting (for example when trying to study cellular processes such as signaling and protein trafficking).

In 2006, a novel chemically induced dimerization (CID) system was developed to enable the selective depletion PIP_2_ at the PM without activating PLC or other downstream pathways [33]. This CID system exploits the ability of two protein domains, one from FK506 binding protein (FKBP) and one from mTOR (mechanistic target of rapamycin) FKBP-rapamycin binding (FRB) to heterodimerize upon the addition of rapamycin or a chemical analog iRap (for review see [34]). By fusing the yeast inositol phosphatase (Inp54p) to FKBP (Inp54p-FKBP) and the FRB domain to a PM anchor Lyn_11_ (Lyn_11_-FRB), Suh and colleagues were able to rapidly recruit and concentrate Inp54p at the PM upon the addition of iRap [33]. Using this CID system the authors elegantly identified that the depletion of PIP_2_ alone, upon recruitment of Inp54p-FKBP to the PM, is sufficient to result in M-channel closure [33].

Similar CID approaches have since been used to examine a variety of biological processes and they provide an exciting tool for studying signaling pathways [35]. In this study, we use a CID system [36] to examine whether PIP_2_ and/or PI(4)P play a role in the anterograde trafficking and function of the Q1/E1 channel complex that underlies *I*_Ks_.

## Materials and methods

### Chemically induced dimerization (CID) system and other constructs

Constructs that were tethered to cell membranes were linked to the FRB domain. LYN_11_-FRB constructs are tethered to the PM by a LYN_11_ sequence (GCIKSKGKDSA): Untagged LYN_11_-FRB (LYN_11_-targeted-FRB) (20147, Addgene; deposited by Professor Tobias Meyer), LYN_11_-FRB-CFP (38003, Addgene) and LYN_11_-FRB-mCherry (38004, Addgene; both deposited by Professor Robin Irvine) [36]. Tgn38-FRB and Tgn38-FRB-CFP are tethered to the *trans*-Golgi network by the integral *trans*-Golgi network protein Tgn38 (a gift from Professor Tamas Balla, National Institute of Child Health and Human Development, National Institutes of Health, Bethesda, United States) [37].

FKBP-containing constructs were those that included phosphatase domains: Pseudojanin (PJ) (37999), PJ-SAC (38000), PJ-INPP5E (38001) and PJ-DEAD (38002) (all from Addgene; all deposited by Professor Robin Irvine) [36]. Each of these constructs consists of a red fluorescent protein (RFP) domain, SAC1 domain, INPP5E domain and an FKBP domain. The SAC1 domain of these constructs encodes the SAC1 phosphatidyl phosphatase, which dephosphorylates phosphatidylinositols at the 4-position, but has no activity against PIP_2_. The INPP5E domain encodes an inositol 1,4,5-trisphosphate (IP_3_) 5-phosphatase, which dephosphorylates PIP_2_ on the 5-position. In PJ both phosphatase domains are active, whilst PJ-SAC contains an inactivating mutation (Asp1263Ala) in the INPP5E domain and PJ-INPP5E contains an inactivating mutation (Cys779Ser) in the SAC1 domain. Therefore, PJ-SAC dephosphorylates only PI(4)P at the 4-position, and PJ-INPP5E dephosphorylates only PIP_2_ at the 5-position. PJ-DEAD contains both inactivating mutations (Asp1263Ala and Cys779Ser). Hence, PJ-DEAD is used as a control construct [36].

The VSV-KCNE1-KCNQ1 (VSV-E1-Q1) construct (a gift from Dr Jean Mérot (Institut du Thorax, Université de Nantes, France)) consists of the VSV epitope fused to the extracellular (N-terminal) side of KCNE1. The C-terminal end of KCNE1 is fused to the N-terminal end of KCNQ1 [38, 39]. The VSV epitope is used for detecting the cell-surface expression of the KCNQ1-KCNE1 channel complex [39]. Human KCNJ2 (Kir2.1) was a gift from Professor Yoshihiro Kubo [40]. Human KCNH2 isoform A (Kv11.1/hERG1a) was a gift from Professor Asipu Sivaprasadarao (University of Leeds, U.K.). KCNQ1, KCNQ1-GFP, KCNQ1 Glu261Asp mutant (KCNQ1-E261D), KCNE1 and DsRed2-ER are as described in [26]. Tubby-YFP was used as previously described [41]. The SAR1 His79Gly mutant (SAR1-H79G) was a gift from Professor Philip Wedegaertner (Thomas Jefferson University, Philadelphia, USA) [42].

### Cell culture and cell lines

Human Embryonic Kidney-293 (HEK293) cells were a gift from Professor Lily Jan (Howard Hughes Medical Institute, San Francisco, USA). Chinese Hamster Ovary-K1 (CHO-K1) cells were purchased from the European Collection of Authenticated Cell Cultures (supplied by Sigma (85051005)). Both cell lines were cultured as previously described [26, 43]. Fugene HD (E2311, Promega) and Lipofectamine 2000 (11668019, Thermo Fisher Scientific) were used for transient transfection of cells according to the manufacturer’s instructions. Stable HEK293 cell lines expressing KCNH2 (Kv11.1; hERG1a; HEK-*I*_Kr_), KCNJ2 (Kir2.1; HEK-*I*_K1_) and KCNQ1-GFP/KCNE1 (HEK-*I*_Ks_) were generated using previously described approaches [44].

### In-cell/on-cell western assays

24-well plates (CELLSTAR, Greiner Bio-One Ltd) were coated with poly-L-lysine (P4707, Sigma Aldrich) before HEK293 cells were seeded at 30% confluence. After 2 days, cells were transfected using Lipofectamine 2000. Cells were transfected with a total of 2 μg cDNA per well. In each well, 1 μg VSV-E1-Q1 was used, and the remaining 1 μg was either SAR1-H79G, KCNQ1-E261D or a combination of 500 ng PJ/PJ-SAC/PJ-DEAD and 500 ng LYN_11_-FRB/Tgn38-FRB. For control wells, and those destined for the addition of wortmannin (1232, Tocris Bioscience) or brefeldin A (B7651, Sigma Aldrich), 1 μg pcDNA3.1 (empty vector) was co-transfected with VSV-E1-Q1.

For the in-cell western assay, cells were washed with PBS^+^ (PBS supplemented with 1 mM MgCl_2_ & 0.1 mM CaCl_2_) then fixed using 3.7% formaldehyde solution (PBS^+^ + formaldehyde) for 20 minutes. After fixing, cells were washed with PBS^+^, and then permeabilized with 0.1% triton-X100 solution (PBS^+^ + triton-X100). Cells were washed with PBS^+^ and incubated in cell culture media for 30 minutes. Primary antibody (Ab) was added (α-KCNQ1; sc-20816, Santa Cruz) in 200 μl cell culture media/well at a 1:1000 dilution, and the cells incubated on a rocking platform at room temperature for 1 hour. Cells were washed with PBS^+^ before adding secondary Ab (α-Rabbit Dylight 800; 5151S, New England Biolabs) in 500 μl cell culture media/well at a 1:1000 dilution. During secondary Ab incubation, cells were protected from light and incubated on a rocking platform at room temperature for 1 hour. Cells were then washed 3 times with PBS^+^, and 500 μl PBS^+^ was added to each well for visualisation.

For the on-cell western assay, cells were either incubated in humidified cell culture incubator at 37°C throughout the assay [37°C live] or were fixed after a 1-hour incubation with the primary Ab at 4°C [4°C fixed] as indicated in the figure legends. 37°C live assay: Primary Ab (α-VSV; V5507, Sigma) was added in 200 μl cell culture media at a 1:500 dilution for 1 hour at 37°C. Cells were then washed three times for 10 mins in cell culture media. Secondary Ab was then added (α-mouse DyLight800, 5257S, New England Biolabs) in 500 μl cell culture media at a 1:1000 dilution for 1 hour. Cells were washed twice with PBS^+^, and 500 μl PBS^+^ was added to each well for visualisation. 4°C fixed assay: Primary Ab (α-VSV) was added in 200 μl ice-cold cell culture media at a 1:500 dilution for 1 hour at 4°C. Cells were then washed once in ice-cold cell culture media and twice in ice-cold PBS^+^. Cells were then fixed using 3.7% formaldehyde for 20 minutes. After fixing, cells were washed with PBS^+^, and secondary Ab was added (α-mouse DyLight800) in 500 μl cell culture media at a 1:1000 dilution for 1 hour at RT. Cells were washed twice with PBS^+^, and 500 μl PBS^+^ was added to each well for visualisation.

Plates were visualised using a LI-COR Odyssey Plate Reader. To correct for any background signal not related to specific channel staining, the mean intensity of the wells that were untransfected was subtracted from the intensity of the wells expressing the channel. To normalise the infrared signals between assays, the mean intensity values from the various transfection conditions were normalised to the mean intensity values from the cells transfected with VSV-E1-Q1 + pcDNA3.1.

Rapamycin (5 μM) (sc-3504, Santa Cruz) was added, either 1 hour or 24 hours before the start of the assay. Wortmannin (10 μM) was added 1 hour prior to the start of the assay, and brefeldin A (5 μM) was added 24 hours before starting the assay.

### Electrophysiology

Whole-cell patch clamp was used to record ionic currents at room temperature using an Axopatch 200B amplifier (Axon Instruments). Transiently transfected cells (500 ng per construct) were identified using epifluorescence and were patch clamped 48–72 hours after transfection. Extracellular bath solution for all experiments with HEK-*I*_Ks_, HEK-*I*_K1_ and CHO-K1 cells contained (mM) 150 NaCl, 5 KCl, 10 HEPES, 2 MgCl_2_ and 1 CaCl_2_ (pH 7.4 with NaOH). Extracellular bath solution for all experiments with HEK-*I*_Kr_ cells contained (mM) 150 NaCl, 4 KCl, 10 HEPES, 1 MgCl_2_, 1.8 CaCl_2_ and 5 glucose (pH 7.4 with NaOH). The intracellular pipette solution contained (mM) 150 KCl, 10 HEPES, 5 EGTA, 2 MgCl_2_, 1 CaCl_2_ and 5 (Na)_2_ATP (pH 7.2 with KOH). Pipette resistance, when pipettes were filled with intracellular solution, was 2–3 MΩ. Pipette tips were coated with SigmaCote (SL2, Sigma) after being filled with intracellular solution, to reduce pipette capacitance. After seal formation and successful rupture of the membrane patch, cells were dialysed for 2 minutes before recording. In all experiments, R_series_ was compensated for by 70% using the amplifier circuitry. The voltage protocols used are indicated in each figure. The liquid junction potentials for both combinations of solutions were relatively small (+4.3 and +4.4 mV respectively) and therefore post-recording adjustments of membrane potential were not performed. In experiments involving the perfusion of rapamycin, extracellular solution containing rapamycin (5 μM) was perfused for 2 minutes, after which perfusion was then returned to rapamycin-free extracellular solution.

Data were analysed using Clampfit (Molecular Devices, CA, U.S.A) and GraphPad Prism (GraphPad Software, Inc., San Diego, CA, U.S.A). Current density (CD) was calculated by normalising the peak current value at the end of each activating pulse to the cell capacitance (nA/pF). Peak tail current density (PTCD) was calculated by normalising the peak tail current value at the start of the repolarising pulse to cell capacitance (pA/pF).

### Confocal microscopy

Live cells transiently transfected with 500 ng of each construct were imaged 48–72 hours after transfection using a Zeiss LSM510 confocal microscope (Mark 4; Carl Zeiss, Oberkochen, Germany) and a Plan-Apochromat 63x oil lens objective. GFP was excited with a multiline argon laser (wavelength 488 nm) and RFP was excited using a helium/neon laser (wavelength 543 nm). Tubby-YFP was imaged using laser settings and filters optimal for excitation of GFP in order to reduce potential cross-talk with RFP. GFP/YFP and RFP/DsRed were excited sequentially to minimise potential crosstalk. Images were median-filtered, converted to RGB format and saved in a JPEG/TIFF format. Colocalisation analysis was performed as described in [45].

### Statistical analysis

Data are expressed as mean ± S.E.M. Statistical analysis was performed using GraphPad Prism. A Student’s t-test (*P* <0.05) was used to determine statistical significance for single comparisons, and a one-way ANOVA (*P* <0.05), with Bonferroni’s multiple comparison test, was used to determine significance when multiple groups were compared.

## Results

### Mutations in a PIP_2_ binding domain in KCNQ1 lead to increased retention of the channel complex in the ER

**Fig 1:**
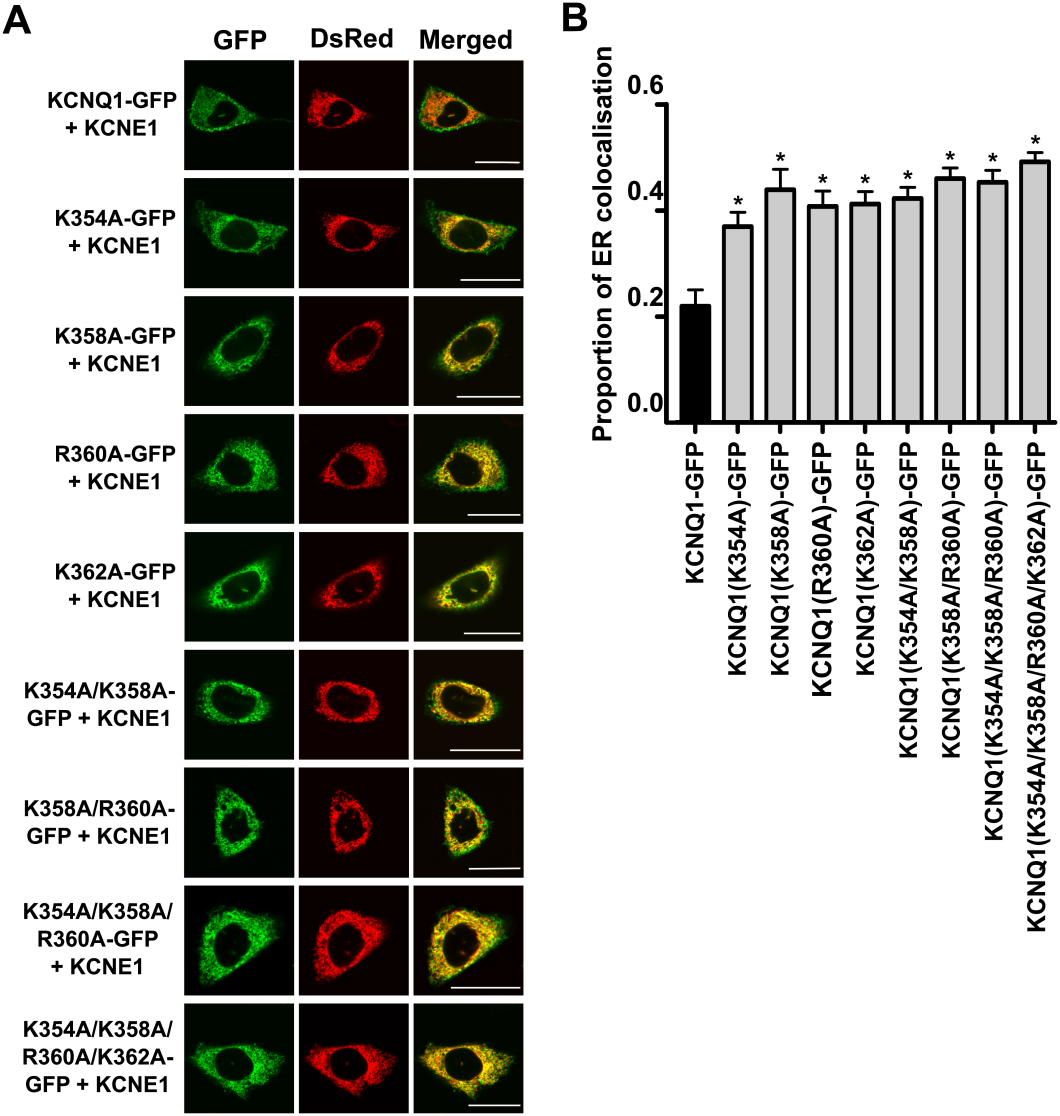
Charge-neutralising mutations in a PIP_2_-binding region in the proximal C-terminus of KCNQ1 increase retention of the channel complex in the ER. **A**. Representative confocal images of the localisation of WT and mutant KCNQ1-GFP channel complexes in CHO-K1 cells. The left panel shows the GFP-tagged KCNQ1 subunit (GFP), the middle panel shows DsRed2-ER, an ER marker, and the right panel shows the merged images. In the merged image panel, the presence of yellow indicates colocalisation between KCNQ1-GFP and DsRed2-ER. Top row: WT KCNQ1-GFP with KCNE1. Lower rows: Localisation of the mutant KCNQ1 channels investigated (all n = 25, except KCNQ1(R360A-GFP), where n = 24). Scale bar indicates 20 μm. **B**. Quantified data showing the proportion of ER colocalisation of the WT and mutant KCNQ1-GFP subunits (in all cases KCNE1 was co-expressed). Data are presented as mean ± S.E.M. * indicates significant difference (*P* <0.05) from control (KCNQ1-GFP + KCNE1) value.

We previously identified charged residues in the proximal C-terminus of KCNQ1 important for phosphoinositide binding [19]. Subsequently, we examined whether the mutation of these residues in KCNQ1-GFP (co-transfected with KCNE1) affected channel trafficking and retention in the ER in transiently transfected CHO-K1 cells. We found that these mutants displayed greater ER retention than the wild-type (WT) channel (Fig 1). This raised the question of whether anionic phospholipid binding was important for the trafficking of KCNQ1 in the secretory pathway. To explore this in a more definitive manner we employed a CID system, described in the materials and methods section, specifically to examine the roles played by PIP_2_ and PI(4)P in Q1/E1 channel trafficking and function.

### Characterisation of the CID system using a specific PIP_2_ reporter

**Fig 2:**
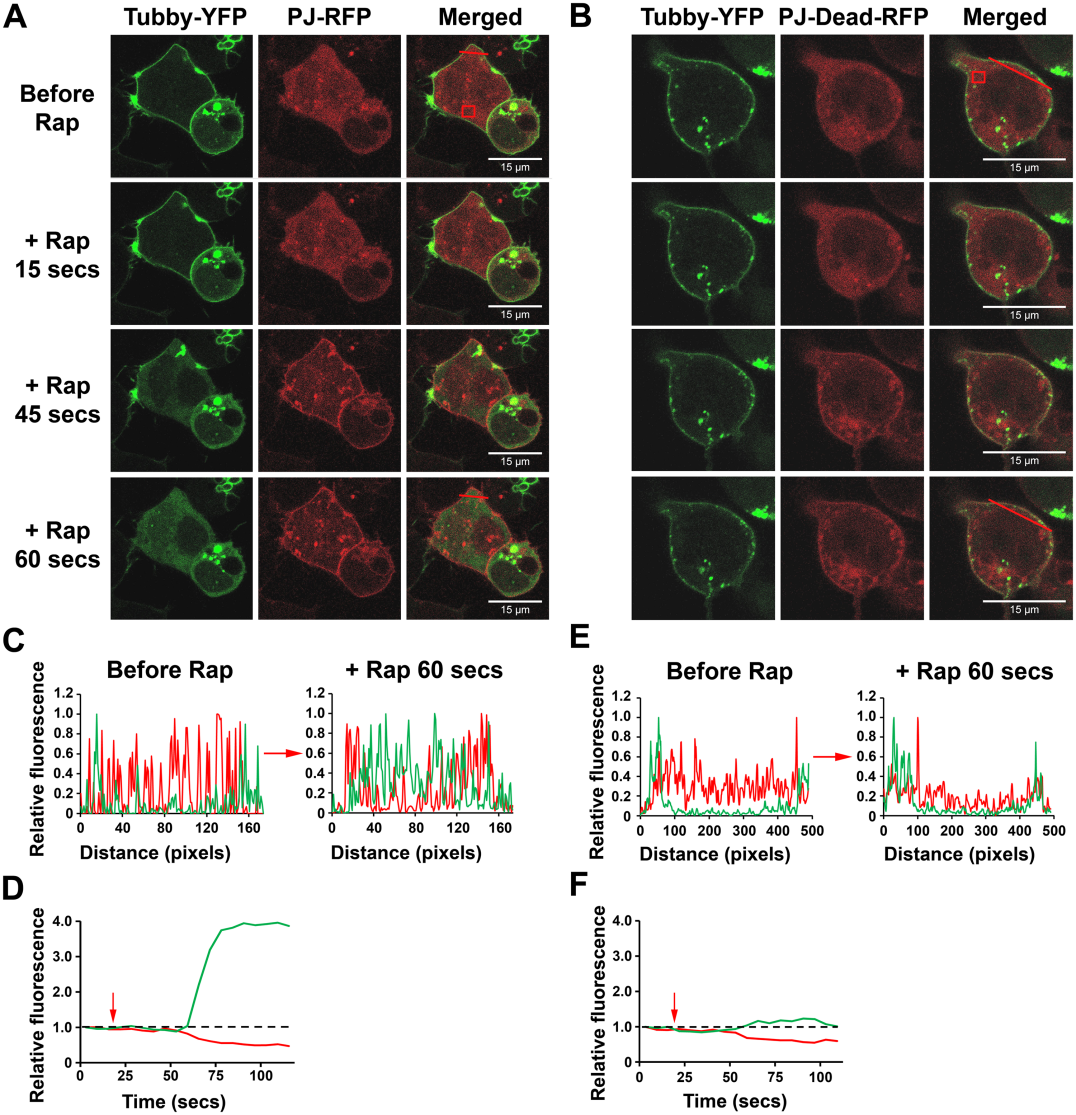
The localisation of the PIP_2_ sensor, Tubby-YFP, in HEK293 cells before and after the rapamycin-induced recruitment of PJ or PJ-DEAD to the PM. The rapamycin (Rap)-induced dimerization of PJ and PJ-DEAD with PM-localised LYN_11_-FRB was investigated in transiently transfected HEK293 cells. Tubby-YFP localises to the PM in the presence of a sufficient PIP_2_ concentration. **A** and **B**. Top panel: Before rapamycin addition. Lower panels: Increasing time after addition of rapamycin showing the increased PM signal of PJ and PJ-DEAD. Upon recruitment of PJ to the PM, Tubby-YFP redistributes from the PM to the cytosol (**A**). Upon recruitment of PJ-DEAD to the PM, Tubby-YFP remains PM-localised (**B**). Scale bar indicates 15 μm. **C–F**. Quantified line (**C** and **E**) and box plots (**D** and **F**) from the indicated red lines and boxed areas in **A** and **B** (located in the top right hand merged panels), highlighting translocation of Tubby-YFP to the cytosol when PJ (**C** and **D**) but not PJ-DEAD (**E** and **F**) is recruited to the PM. Red arrows indicate rapamycin addition.

Previously we developed a specific fluorescent probe that is able to monitor and report changes in PIP_2_ levels at the PM, named Tubby-YFP [41]. Using Tubby-YFP we monitored whether the CID system was working as expected. PJ-DEAD or PJ were transiently expressed with LYN_11_-FRB and Tubby-YFP in HEK293 cells, and imaged using confocal microscopy. When rapamycin (5 μM) was added to cells expressing PJ-DEAD or PJ with LYN_11_-FRB and Tubby-YFP, both PJ-DEAD and PJ translocated from the cytosol/nucleus to the PM in 30–45 seconds (Fig 2A and 2B). In cells expressing PJ, Tubby-YFP underwent a rapid redistribution after the translocation of PJ to the PM (Fig 2A, 2C and 2D). At 45 seconds after rapamycin addition, Tubby-YFP had partially moved from the PM to the cytosol, and at 60 seconds it showed a complete redistribution (Fig 2A, 2C and 2D). In contrast, in cells expressing PJ-DEAD there was no movement of Tubby-YFP after translocation of PJ-DEAD to the PM (Fig 2B, 2E and 2F). The redistribution of Tubby-YFP from the PM to the cytosol when PJ, but not PJ-DEAD, was recruited to the PM indicates that the CID system was working as expected.

### PIP_2_/PI(4)P modulation of *I*_K1_ and *I*_Kr_ channel function

**Fig 3:**
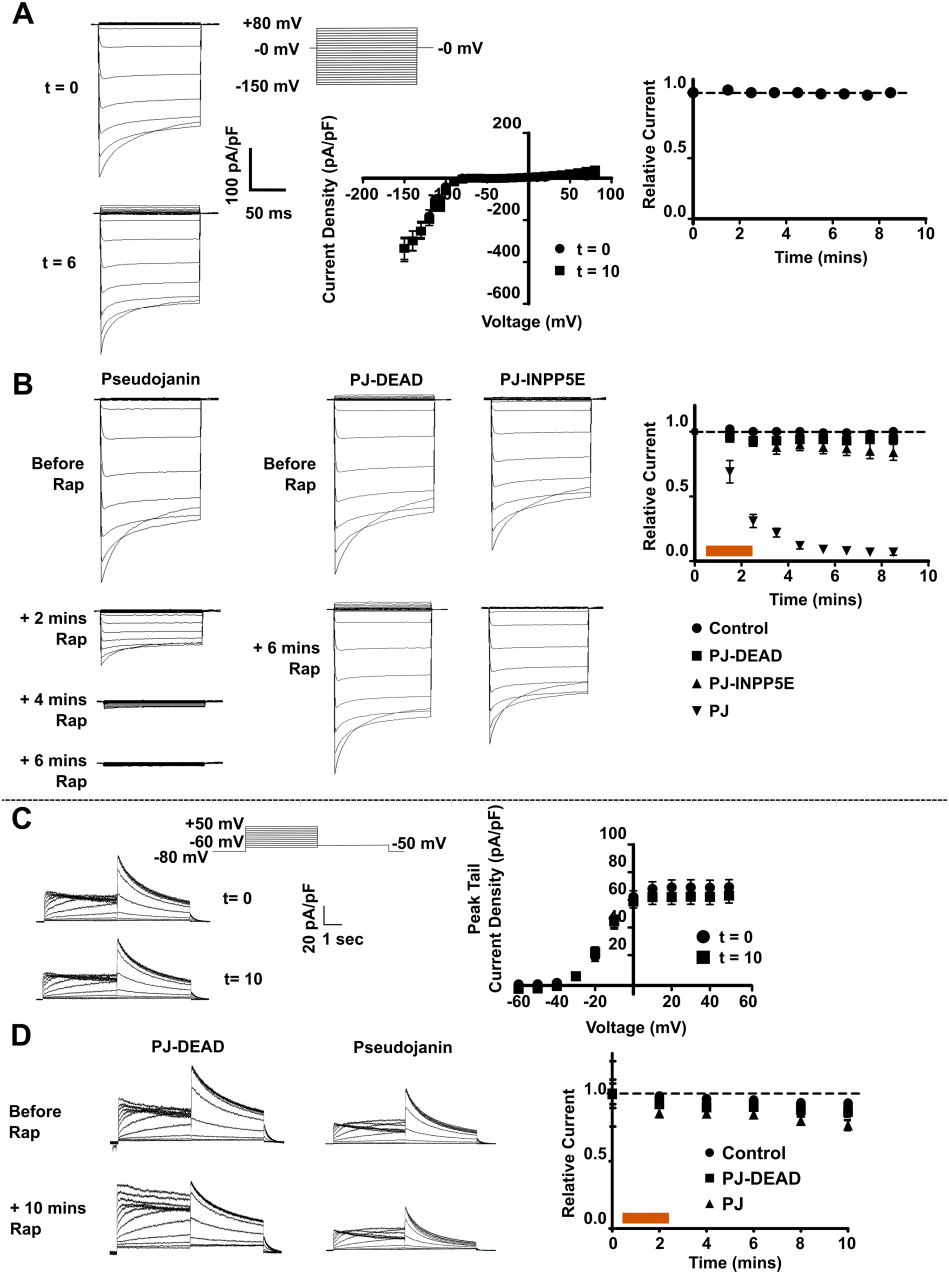
Rapamycin-induced recruitment of lipid-depleting constructs reduces *I*_K1_ but not *I*_Kr_. **A**. Representative whole-cell current traces (t = 0 and 6 minutes), CD (t = 0 and 10 minutes) and relative current (at -150 mV) (normalised to 0 minutes) from HEK-*I*_K1_ cells. **B**. Representative current traces and relative current (at -150 mV) (normalised to 0 minutes) from HEK-*I*_K1_ cells expressing PJ, PJ-DEAD or PJ-INPP5E with LYN_11_-FRB, before and 6 minutes after rapamycin (5 μM) perfusion (orange bar). PJ (n = 9), PJ-DEAD (n = 9) or PJ-INPP5E (n = 11). Relative CD from untransfected HEK-*I*_K1_ cells (at -150 mV) (control; n = 11) is included for comparison. **C**. Representative current traces and PTCD from HEK-*I*_Kr_ cells recorded at t = 0 and 10 minutes. **D**. Representative current traces and relative current (at 0 mV) (normalised to 0 mins) from HEK-*I*_Kr_ cells expressing PJ or PJ-DEAD with LYN_11_-FRB, before and 10 minutes after rapamycin (5 μM) perfusion (orange bar). PJ-DEAD (n = 10) or PJ (n = 10). Relative current (at 0 mV) from untransfected HEK-*I*_Kr_ cells (control; n = 9) included for comparison. Data are presented as mean ± S.E.M. * indicates significant difference (*P* <0.05) from control value.

We next examined the effects of CID-system-mediated depletion of PIP_2_ and PI(4)P at the PM on the cloned equivalents of cardiac currents *I*_K1_ and *I*_Kr_ in HEK293 cell lines stably expressing hKir2.1 (KCNJ2) and Kv11.1 (KCNH2; hERG1a), respectively. In the recording conditions used, the currents were stable for ~10 minutes and did not run down (Fig 3A and 3C). The expression of any combination of the CID constructs prior to the addition of rapamycin did not act to significantly suppress either of the currents (S1 Fig and Fig 3B and 3D). Rapamycin (5 μM) was perfused over HEK-*I*_K1_ cells expressing PJ-DEAD and LYN_11_-FRB for 2 minutes after the completion of the first I-V recording. The inward rectifier current was recorded every minute for 8 minutes. Over this period, there was no change in peak inward current density (CD) (*P* = NS) (Fig 3B). The same experiment was repeated in HEK-*I*_K1_ cells expressing PJ-INPP5E and LYN_11_-FRB, and after 8 minutes there was no change in the peak inward CD (*P* = NS) (Fig 3B). Finally, rapamycin was added to cells expressing PJ and LYN_11_-FRB. After rapamycin (5 μM) perfusion for 2 minutes there was a gradual but complete inhibition of the peak inward *I*_K1_ CD over approximately 6 minutes (*P* <0.05 at +1 min rapamycin, and *P* <0.005 at +2 minutes rapamycin onwards), with only endogenous HEK293 current remaining from 6 minutes (Fig 3B). This current was significantly reduced compared to control (*P* <0.001 across all time points analysed) and PJ-DEAD (*P* <0.001 across all time points analysed).

In an analogous fashion we examined the effects on *I*_Kr_. Rapamycin (5 μM) was perfused over HEK-*I*_Kr_ cells expressing PJ-DEAD and LYN_11_-FRB for 2 minutes after completion of the first I-V recording. Over a period of 10 minutes, there was a small reduction in peak tail current density (PTCD), which was not significant (*P* = NS) and was similar to untransfected cells (Fig 3D). When rapamycin was perfused over HEK-*I*_Kr_ cells expressing PJ and LYN_11_-FRB for 2 minutes, there was a small inhibition of the PTCD (Fig 3D) beyond that seen in untransfected HEK-*I*_Kr_ cells and cells expressing PJ-DEAD and LYN_11_-FRB, but this difference was not significant (*P* = NS). These results highlight that *I*_K1_ is significantly inhibited by dual PIP_2_/PI(4)P depletion whilst *I*_Kr_ is not.

### PIP_2_/PI(4)P depletion strongly modulates Q1/E1 channel function

**Fig 4:**
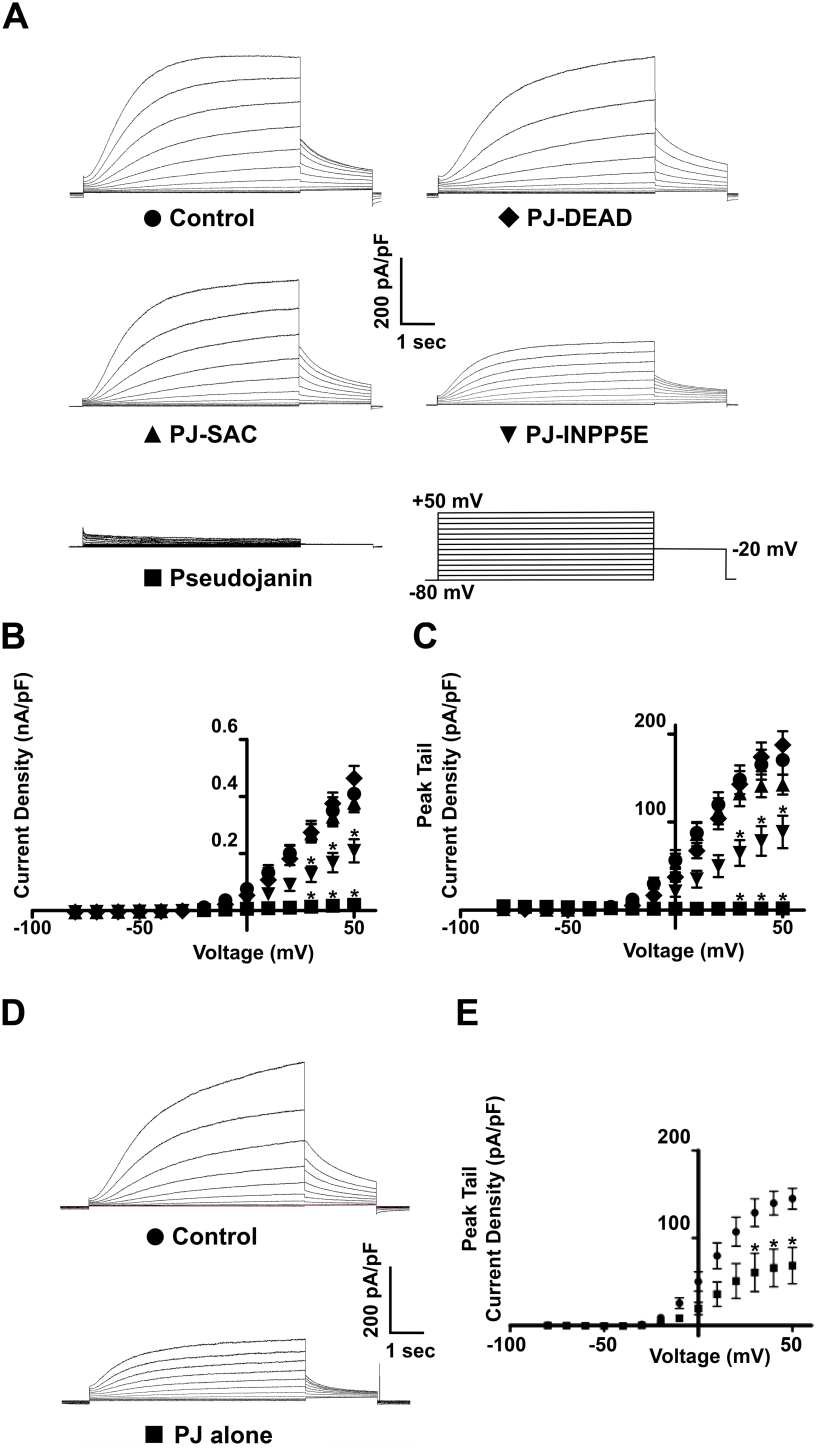
The expression of lipid-depleting dimerization constructs dramatically reduces *I*_Ks_ prior to rapamycin addition. **A**. Representative traces of currents recorded from HEK-*I*_Ks_ cells (control), and HEK-*I*_Ks_ cells transiently expressing PJ-DEAD, PJ-SAC, PJ-INPP5E or PJ with LYN_11_-FRB. Mean CD **(B)** and PTCD **(C)** of currents from HEK-*I*_Ks_ cells transiently expressing PJ (n = 11), PJ-SAC (n = 23), PJ-INPP5E (n = 24) or PJ-DEAD (n = 16) with LYN_11_-FRB. **D**. Representative traces of the effect of PJ expression alone (without LYN_11_-FRB) on *I*_Ks_ in HEK-*I*_Ks_ cells. **E**. Effect of PJ expression alone (without LYN_11_-FRB) on *I*_Ks_ PTCD in HEK-*I*_Ks_ cells (PJ-expressing cells, n = 12; control (untransfected cells), n = 12). Data are presented as mean ± S.E.M. * indicates significant difference (*P* <0.05) from control (untransfected cells) value.

Expression of PJ-DEAD and LYN_11_-FRB had no effect on *I*_Ks_ CD or PTCD in HEK-*I*_Ks_ cells compared to control (untransfected) cells (Fig 4A, 4B and 4C). Next, PJ-SAC, PJ-INPP5E or PJ were transiently expressed with LYN_11_-FRB in HEK-*I*_Ks_ cells, and *I*_Ks_ was recorded in each condition. Expression of PJ-SAC with LYN_11_-FRB had no effect on *I*_Ks_ CD or PTCD (*P* = NS), while expression of PJ-INPP5E with LYN_11_-FRB resulted in a reduction of *I*_Ks_ CD and PTCD by approximately 50% compared to control (*P* <0.005 across all voltages analysed for both CD and PTCD) (Fig 4A, 4B and 4C). PJ-INPP5E also significantly reduced *I*_Ks_ compared to PJ-DEAD (*P* <0.001 across all voltages analysed for both CD and PTCD) (Fig 4A, 4B and 4C). Expression of PJ with LYN_11_-FRB in HEK-*I*_Ks_ cells caused a near complete loss of *I*_Ks_ in the absence of rapamycin application (*P* <0.001 across all voltages analysed for CD and PTCD when compared to control or PJ-DEAD) (Fig 4A, 4B and 4C).

Due to the near complete loss of *I*_Ks_ that was observed in HEK-*I*_Ks_ cells expressing PJ with LYN_11_-FRB, a similar experiment was performed in CHO-K1 cells to rule out cell-type-specific effects. The *I*_Ks_ channel subunits, KCNQ1 and KCNE1, were expressed in CHO-K1 cells with LYN_11_-FRB and PJ-DEAD, and the resulting current was recorded. While expressing PJ-DEAD, *I*_Ks_ current was detected. However, the expression of PJ in place of PJ-DEAD caused a dramatic loss of the current as previously seen in HEK-*I*_Ks_ cells, which was significantly reduced (*P* <0.0001) across all voltages analysed for both CD and PTCD (S2 Fig). A similar suppression of KCNQ1 (Kv7.1) currents occurred if KCNQ1 was expressed in the absence of KCNE1 in CHO-K1 cells (S3 Fig). In addition, when PJ was expressed alone (without LYN_11_-FRB) in HEK-*I*_Ks_ cells there was a substantial, albeit less prominent, reduction (~50%) in current (Fig 4D and 4E).

### The depletion of PIP_2_ and/or PI(4)P at the PM and PI(4)P in the Golgi does not affect the cell surface expression level of KCNQ1

**Fig 5:**
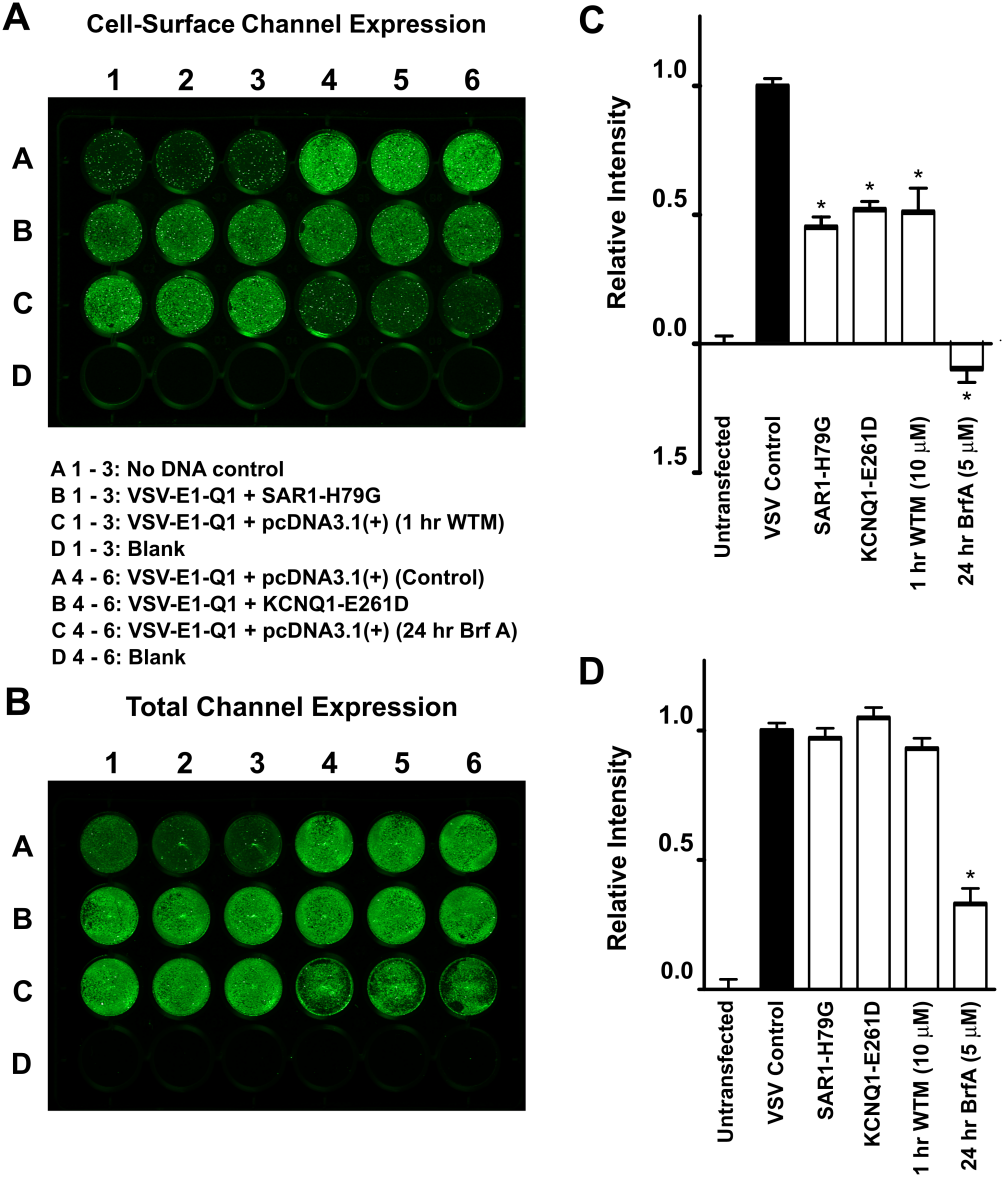
Measuring and manipulating the cell-surface and total expression level of KCNQ1. **A** and **B**. Representative on-cell [37°C live] and in-cell western assays, enabling quantification of the cell-surface and total channel expression of the VSV-KCNE1-KCNQ1 (VSV-E1-Q1) channel, respectively. The VSV-E1-Q1 construct was co-expressed in HEK293 cells with either pcDNA3.1, SAR1-H79G or KCNQ1-E261D. Cells expressing VSV-E1-Q1 with pcDNA3.1 were incubated with either 10 μM wortmannin (WTM) for 1 hour or 5 μM brefeldin A (Brf A) for 24 hours before the start of the assay. Each condition was performed in triplicate. Mean data, normalised to VSV-E1-Q1 + pcDNA3.1 values (VSV Control), from three independent experiments for cell-surface and total channel expression are shown in **C** and **D**, respectively. Data presented as mean ± S.E.M. * indicates significant difference (*P* <0.05) from control (VSV-E1-Q1 + pcDNA3.1) value.

We used in-cell and on-cell western assays to measure the surface expression and trafficking of VSV-E1-Q1. When HEK293 cells transiently expressing VSV-E1-Q1 were incubated for 1 hour with wortmannin (10 μM), which inhibits both phosphatidylinositol 4-kinase (PI4K) and 3-kinase (PI3K) activity at this concentration [46], there was no change in the total VSV-E1-Q1 channel expression. Wortmannin incubation did, however, cause a significant reduction in cell-surface expression of the VSV-E1-Q1 construct (*P* <0.01) (Fig 5). Incubation of VSV-E1-Q1-expressing HEK293 cells with brefeldin A (5 μM) for 24 hours resulted in some cell death. The total cellular expression of VSV-E1-Q1 was significantly reduced (*P* <0.001) after 24 hours brefeldin A incubation, but it is unclear whether this was solely due to the reduction in total cell number or whether there was an actual reduction in VSV-E1-Q1 protein expression (Fig 5D). Brefeldin A incubation also dramatically reduced the cell-surface expression of the VSV-E1-Q1 construct (*P* <0.001) (Fig 5C).

In addition, the assay was able to report genetically encoded perturbations that might affect channel trafficking. We have previously shown that the KCNQ1 mutant, KCNQ1-E261D, is retained in the ER and that this behaviour is dominant, i.e. co-expression with WT leads to retention of both mutant and WT [26]. Overexpression of KCNQ1-E261D led to reduction in cell-surface but not total expression (Fig 5). Furthermore, expression of SAR1-H79G, which disrupts ER export, also decreased cell surface but not total expression (Fig 5).

**Fig 6:**
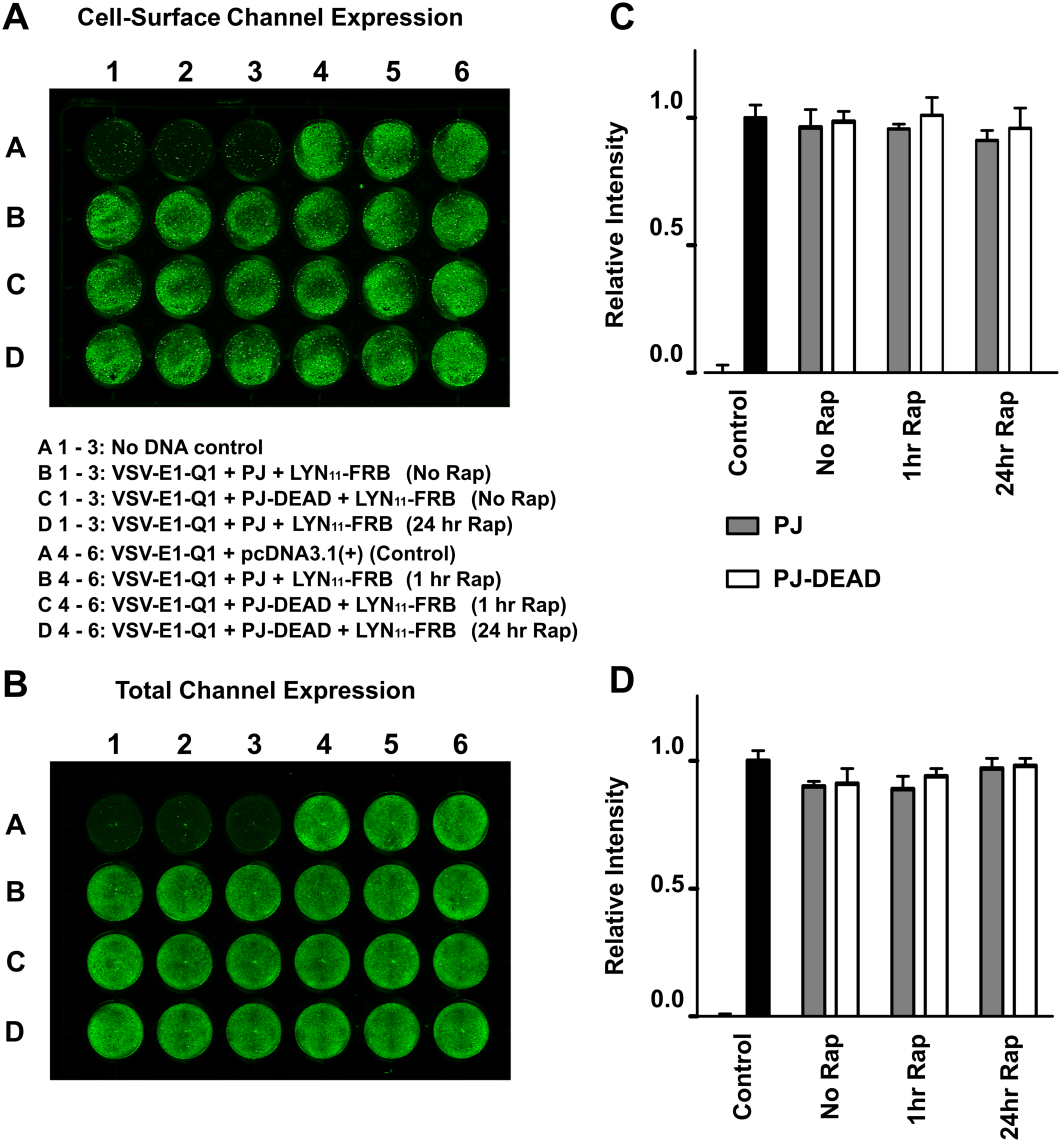
Acute or long-term depletion of PIP_2_ at the PM does not affect the cell-surface or total expression level of KCNQ1. **A** and **B**. Representative on-cell [37°C live] and in-cell western assays, enabling quantification of the cell-surface and total channel expression, respectively. The VSV-E1-Q1 construct was co-expressed in HEK293 cells with pcDNA3.1, or with LYN_11_-FRB and PJ or PJ-DEAD. Cells expressing LYN_11_-FRB with either PJ or PJ-DEAD were incubated in the absence of 5 μM rapamycin (Rap), or in the presence of Rap for 1 hour or 24 hours as indicated. Each condition was performed in triplicate. Mean data, normalised to VSV-E1-Q1 + pcDNA3.1 values (VSV Control), from three independent experiments for cell-surface and total channel expression are shown in **C** and **D**, respectively. Data presented as mean ± S.E.M. * indicates significant difference (*P* <0.05) from control (VSV-E1-Q1 + pcDNA3.1) values.

We next tested whether acute or longer term depletion of PIP_2_/PI(4)P at the PM can affect channel trafficking. To do this we transiently expressed VSV-E1-Q1, LYN_11_-FRB and either PJ-DEAD or PJ in HEK293 cells and recruited the lipid depleting constructs to the PM by adding rapamycin for 1 or 24 hours prior to the start of the assay. In the absence of rapamycin the expression of PJ-DEAD or PJ did not have an effect (*P* = NS) on cell-surface or total expression of the channel (Fig 6). In addition, the application of rapamycin for either 1 or 24 hours, and therefore induction of PIP_2_/PI(4)P depletion at the PM, had no effect (*P* = NS) on cell-surface or total expression of the channel (Fig 6).

**Fig 7:**
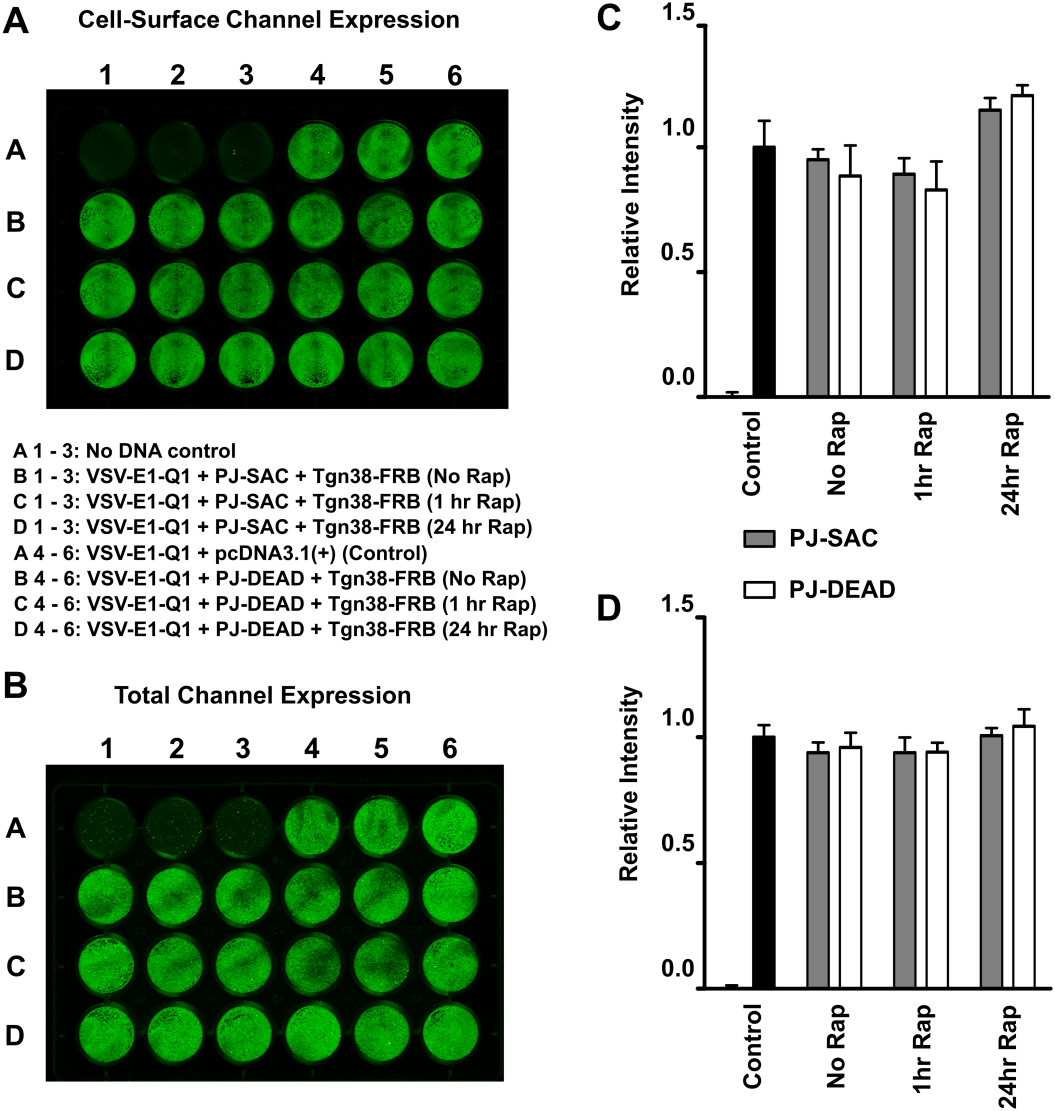
Depletion of PI(4)P at the Golgi does not affect the cell-surface or total expression level of KCNQ1. **A** and **B**. Representative on-cell [4°C fixed] and in-cell western assays, enabling quantification of the cell-surface and total cellular expression, respectively. The VSV-E1-Q1 construct was co-expressed in HEK293 cells with pcDNA3.1, or with Tgn38-FRB and PJ-SAC or PJ-DEAD. Cells expressing Tgn38-FRB with either PJ-SAC or PJ-DEAD were incubated in the absence of 5 μM rapamycin (Rap), or in the presence of Rap for 1 hour or 24 hours as indicated. Each condition was performed in triplicate. Mean data, normalised to VSV-E1-Q1 + pcDNA3.1 values (VSV Control), from three independent experiments for cell-surface and total channel expression are shown in **C** and **D**, respectively. Data presented as mean ± S.E.M. * indicates significant difference (*P* <0.05) from control (VSV-E1-Q1 + pcDNA3.1) values.

PI(4)P is concentrated in the Golgi and is thought to be important for transport of specific cargo from the Golgi to the PM [37]. To investigate whether Golgi-localised PI(4)P plays a role in Q1/E1 trafficking we transiently expressed VSV-E1-Q1, Tgn38-FRB, and either PJ-DEAD or PJ-SAC in HEK293 cells. In the absence of rapamycin, and therefore Golgi recruitment, the expression of PJ-DEAD or PJ-SAC did not have an effect (*P* = NS) on cell-surface or total expression of the channel (Fig 7). Similarly, the addition of rapamycin, and therefore depletion of Golgi-localised PI(4)P, for either 1 or 24 hours failed to effect (*P* = NS) the total or cell-surface expression level of the channel (Fig 7).

## Discussion

### Differential sensitivity of cardiac ion channels to PIP_2_

In this study, Q1/E1 channel function was sensitive to the depletion of PIP_2_. Even in the absence of rapamycin (the dimerizing agent) the co-expression of PJ and LYN_11_-FRB dramatically reduced *I*_Ks_. We reason below that this occurs because of a high sensitivity of the channel to changes in PM PIP_2_ levels, which are reduced by the basal constitutive phosphatase activity of the CID system. The *I*_Ks_ channel β-subunit, KCNE1, confers altered sensitivity to PIP_2_ [47]. As such, we additionally investigated the regulation of the homomeric KCNQ1 channel. The loss of this current when expressing PJ and LYN_11_-FRB, in the absence of rapamycin, also indicates that the homomeric KCNQ1 channel has high sensitivity to PM PIP_2_ depletion, which supports the findings of Li and colleagues [47].

For comparison, we investigated the PIP_2_ regulation of Kir2.1 and Kv11.1 channels (the pore-forming subunits of the *I*_K1_ and *I*_Kr_ currents, respectively) as these are also present in ventricular myocytes and contribute to ventricular repolarisation. In contrast to *I*_Ks_, the co-expression of PJ with LYN_11_-FRB, in the absence of rapamycin, had no effect on either of the currents. In HEK-*I*_K1_ cells, recruitment of PJ to the PM caused a gradual and complete inhibition of *I*_K1_, and in HEK-*I*_Kr_ cells PJ recruitment caused a small (but not significant) reduction in *I*_Kr_ over the course of the experiment. Thus, Kv11.1/*I*_Kr_ is relatively insensitive to PIP_2_/PI(4)P depletion, whilst Kir2.1/*I*_K1_ is sensitive. Our findings agree with those reported by Kruse and colleagues, who showed that Kv11.1 is insensitive to joint depletion of PIP_2_ and PI(4)P [32]. Prior studies have reported that Kv11.1 is regulated by PIP_2_, although the effects on channel function are modest [48, 49]. Recently it has been shown that the co-expression of KCNE2 [32] or the G_q/11_-coupled Muscarinic-1 receptor [50] with Kv11.1 confers increased sensitivity to PIP_2_. This suggests that the presence of accessory subunits (such as the KCNE family) or G_q/11_-coupled GPCRs, which are expressed in ventricular myocytes, may enhance the PIP_2_ sensitivity of the *I*_Kr_ channel complex.

The complete loss of *I*_Ks_ even before rapamycin addition indicates that this channel is extremely sensitive to PIP_2_ depletion and suggests that the channel complex has a relatively low apparent affinity for PIP_2_. In contrast, Kir2.1/*I*_K1_ was only significantly inhibited when PJ was recruited to the PM and both PIP_2_ and PI(4)P were depleted. The inability of PJ-INPP5E recruitment to inhibit Kir2.1/*I*_K1_ could have been because the level of PIP_2_ depletion achieved was not high enough to inhibit channel function, or alternatively it could be because PI(4)P is equally able to activate the channel in the absence of PIP_2_ (especially since PJ-INPP5E converts PIP_2_ to PI(4)P). A recent study directly addressed this question and found that the application of PI(4)P, in giant excised patches, does not activate Kir2.1 [51]. Therefore, we feel that the differences in the ability of PJ and PJ-INPP5E to inhibit Kir2.1/*I*_K1_ directly reflect how strongly they can deplete PIP_2_. In comparison to *I*_Ks_, the inhibition of Kir2.1/*I*_K1_ channel activity requires a greater reduction in PIP_2_. This implies that Kir2.1 has a higher relative apparent affinity for PIP_2_. To our knowledge the relative affinities of Q1/E1 and Kir2.1 for PIP_2_ have not been directly compared (in the same study). However, Kir2.1 has been postulated to have an approximately seventeen-fold higher affinity than KCNQ2 for PIP_2_ [32]. Based on our findings we speculate that the activity of *I*_Ks_, but not *I*_K1_ or *I*_Kr_, is likely to be strongly affected by even small fluctuations in the levels of PIP_2_. This is similar to our previous observations regarding the PIP_2_ sensitivity of ATP-sensitive potassium channels [52], and is consistent with the findings by Kruse and colleagues that only a specific subset of ion channels are highly sensitive to PIP_2_ depletion [32, 53].

### Drawbacks of the CID system

Manipulation of cellular PIP_2_ levels using methods such as excised-patch recordings [16], G_q/11_-coupled receptor activation [54] and exogenous application of PIP_2_ analogues [25] give insights into the dependence of the Q1/E1 channel on PIP_2_ for function. However, what these experimental methods do not allow is a direct, isolated and specific manipulation of PIP_2_ levels within the cell. In theory, the CID system should overcome these issues, and it has indeed been used to investigate a range of ion channels including the Q1/E1 channel and other members of the KCNQ family [32]. However, when we expressed PJ or PJ-INPP5E with LYN_11_-FRB, inhibition of *I*_Ks_ occurred even before rapamycin was added.

Could a low level of lipid depletion at the PM occur by expressing active lipid-depleting constructs, even in the absence of rapamycin? The phosphatase domains of the lipid-depleting constructs are constitutively active, which could confer a low level of cellular PIP_2_ depletion (and/or PI(4)P in the case of PJ and PJ-SAC, respectively). In our imaging experiments it is clear that the lipid-depleting constructs are expressed throughout the cytosol. The cytosolic expression of the phosphatase constructs could allow a low level of lipid depletion by constructs that are coincidentally close to the PM, as has been discussed previously [35]. Even without LYN_11_-FRB, the expression of PJ in HEK-*I*_Ks_ cells caused a 50% reduction of current.

Interestingly, the fact that LYN_11_-FRB expression confers increased inhibition of *I*_Ks_ indicates that there may be a low level interaction between these two constructs, possibly between the FRB and FKBP domains in the absence of rapamycin. A 2.2 Angstrom resolution structure of the FRB-rapamycin-FKBP complex showed multiple residues involved in the protein-protein interaction [55]. A transient and weak interaction could somewhat concentrate the lipid-depleting constructs at the PM. Overall, our findings highlight that the constitutive phosphatase activity of the CID system is an inherent problem when trying to study highly sensitive PIP_2_ effectors, such as the Q1/E1 channel.

### PIP_2_ and PI(4)P do not regulate the cell-surface expression level of Q1/E1

The observation that the CID system was not optimal for investigation of Q1/E1 channel function does not preclude using it to study the role of PIP_2_ and other anionic phospholipids in channel trafficking. To calibrate the in-cell and on-cell western assays a number of positive control experiments were performed. These were known to affect broad trafficking pathways or specifically channel surface expression. The expression of the SAR1 mutant, SAR1-H79G, and the KCNQ1 dominant negative (DN) mutant, KCNQ1-E2691D, both caused a reduction in the cell-surface expression of the VSV-E1-Q1 construct whilst preserving total channel expression. SAR1-H79G has a DN effect over WT SAR1 and prevents vesicle trafficking between the ER and Golgi [42], explaining the reduction in VSV-E1-Q1 cell-surface expression. KCNQ1-E261D is retained in the ER and has a DN effect over the trafficking of the WT channel [26, 56]. These cellular manipulations indicate that the in-cell/on-cell western assays can faithfully report changes in channel trafficking to the PM.

To find out if acute or long-term depletion of PIP_2_ and/or PI(4)P at the PM affected trafficking of the Q1/E1 channel, cells expressing the lipid-depleting constructs were incubated with or without rapamycin for 1 hour or 24 hours. None of these conditions resulted in any changes in the cell-surface or total expression of the channel, suggesting that the reduction of PIP_2_ and/or PI(4)P at the PM does not affect trafficking or expression. PI(4)P is present at the Golgi in higher concentrations than the rest of the cell, and is suggested to be involved in the anterograde trafficking of proteins destined for the PM. Thus, we depleted PI(4)P at the Golgi using PJ-SAC expressed with Tgn38-FRB [37, 57]. We found that there was no change in channel cell-surface or total expression when PJ-SAC was recruited to the Golgi with either 1 or 24 hour rapamycin incubation, despite Golgi recruitment of CID constructs (S4 Fig).

In contrast, wortmannin incubation (for 1 hour) reduced the cell-surface expression of VSV-E1-Q1 (by ~50%) without altering total channel expression. Given that the depletion of PIP_2_ and/or PI(4)P (upon recruitment of PJ to the PM and PJ-Sac to the Golgi) did not alter Q1/E1 trafficking we feel that it is unlikely that the effects of wortmannin are mediated through PI4K inhibition. At both high and low concentrations wortmannin is a potent inhibitor of PI3Ks [58]. PI3Ks control the production of a number of phosphoinositides including phosphatidylinositol-3-phosphate (PI(3)P), phosphatidylinositol-3,4-bisphosphate (PI(3,4)P_2_) and phosphatidylinositol-3,4,5-trisphosphate (PI(3,4,5)P_3_) [59]. Wortmannin-mediated disruption of PI3K activity could therefore lead to reduced levels of these phosphoinositides. PI(3)P is highly concentrated on early endosomes and is involved in the recycling of membrane proteins [13], which may explain why the trafficking of the channel is perturbed. Additionally, the reduction of PI(3)P could also lead to reduced levels of phosphatidylinositol-3,5-bisphosphate (PI(3,5)P_2_). Intriguingly, an enhanced production of PI(3,5)P_2_ has been suggested to be central in mediating an increased rate of Q1/E1 channel exocytosis upon activation of the serum and glucocorticoid-inducible kinase 1 [60].

Our findings suggest that the increased degree of channel retention in the ER seen upon charge neutralisation of residues in a PIP_2_ binding site (Fig 1) is not due to perturbed binding to PIP_2_ and or PI(4)P. It is possible that the increases seen in ER channel retention are solely due to non-specific effects of these mutations on protein stability/folding. However, the inhibitory effects of wortmannin on Q1/E1 trafficking highlight that other phosphoinositides, such as PI(3)P and PI(3,5)P_2_, could play a role and this warrants future investigation.

In conclusion, we identify that the Q1/E1 channel does not require PIP_2_ and/or PI(4)P for anterograde trafficking, but is heavily reliant on PIP_2_ for function once at the PM. It has been postulated that the relatively low level of PIP_2_ in the secretory pathway is important for silencing (or activating) specific ion channels whilst in transit [11, 61]. For Q1/E1, our findings support this hypothesis and it is likely that the channel becomes activated by PIP_2_ once it reaches the PM.

## Supporting information

S1 Fig The effect of expressing lipid-depleting constructs on *I*_K1_ and *I*_Kr_. **A**. Representative traces of currents recorded from HEK-*I*_K1_ cells (control), and HEK-*I*_K1_ cells transiently expressing PJ-DEAD, PJ-INPP5E or PJ with LYN_11_-FRB. **B**. Mean CD of currents recorded from untransfected HEK-*I*_K1_ cells (control; n = 11) and HEK-*I*_K1_ cells transiently expressing PJ (n = 9), PJ-INPP5E (n = 11) or PJ-DEAD (n = 9) with LYN_11_-FRB. **C**. Representative traces of currents recorded from HEK-*I*_Kr_ cells (control), and HEK-*I*_Kr_ cells transiently expressing PJ-DEAD or PJ with LYN_11_-FRB. **D**. Mean PTCD of currents recorded from HEK-*I*_Kr_ cells (control; n = 9) and HEK-*I*_Kr_ cells transiently expressing PJ (n = 10) or PJ-DEAD (n = 10) with LYN_11_-FRB. Data are presented as mean ± S.E.M.
(TIF)

S2 Fig The effect of expressing lipid-depleting constructs in CHO-K1 cells expressing *I*_Ks_. **A**. Representative traces of currents recorded from CHO-K1 cells transiently expressing the KCNQ1 and KCNE1 subunits with LYN_11_-FRB and either PJ-DEAD or PJ. **B**. Voltage protocol used to elicit current recorded. **C**. Mean CD (top) and PTCD (bottom) of currents from CHO-K1 cells transiently expressing KCNQ1 and KCNE1 with LYN_11_-FRB and either PJ-DEAD (n = 12) or PJ (n = 11). Data are presented as mean ± S.E.M. An unpaired t-test was performed to determine statistical significance between groups in **C**, at voltages between +30 mV and +80 mV. * indicates significant difference (*P* <0.05) from control (KCNQ1 + KCNE1 + PJ-DEAD) value.
(TIF)

S3 Fig The effect of expressing lipid-depleting constructs in CHO-K1 cells expressing the homomeric KCNQ1 channel. **A**. Representative traces of currents recorded from CHO-K1 cells transiently expressing KCNQ1 with LYN_11_-FRB and either PJ-DEAD or PJ. **B**. Voltage protocol used to elicit current recorded. **C**. Mean CD (top) and PTCD (bottom) of currents from CHO-K1 cells transiently expressing KCNQ1 with LYN_11_-FRB and either PJ-DEAD (n = 11) or PJ (n = 10). Data are presented as mean ± S.E.M. An unpaired t-test was performed to determine statistical significance between groups in **C**, at voltages between +30 mV and +80 mV. * indicates significant difference (*P* <0.05) from control (KCNQ1 + PJ-DEAD) value.
(TIF)

S4 Fig The rapamycin-induced recruitment of PJ to the Golgi. Top panel: The localisation of PJ and Tgn38-FRB in HEK293 cells. The five centre and bottom rows show the same cell at different time points after the addition of rapamycin (5 μM). Scale bar indicates 20 μm.
(TIF)
