# Supplementary figures and images for: Phosphatidylinositol-4,5-bisphosphate is required for KCNQ1/KCNE1 channel function but not anterograde trafficking
Source: PLoS One. 2017 Oct 11;12(10):e0186293. doi: 10.1371/journal.pone.0186293 (PMC5636153; doi:10.1371/journal.pone.0186293)

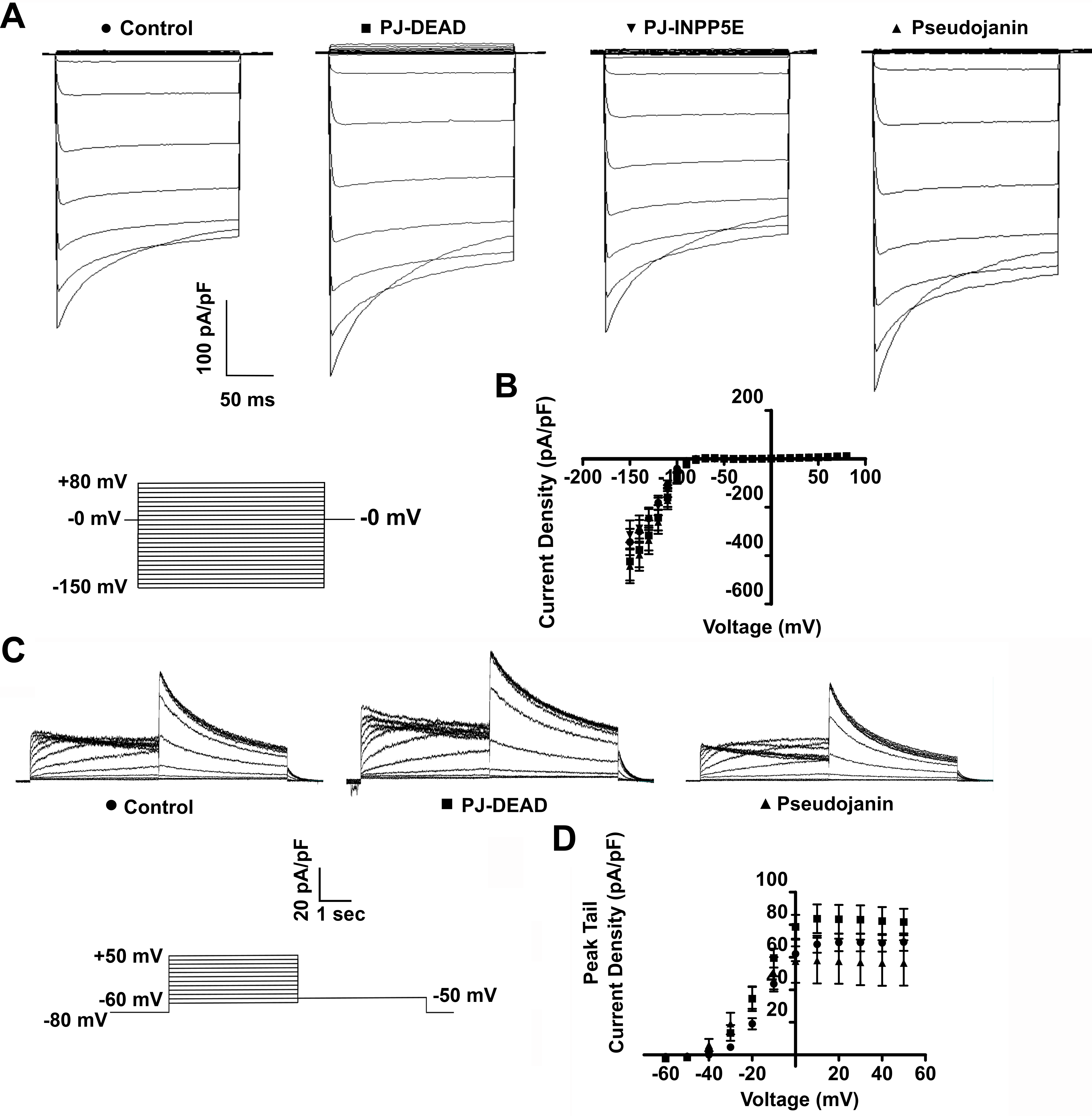

Supplement: S1 Fig — A. Representative traces of currents recorded from HEK-IK1 cells (control), and HEK-IK1 cells transiently expressing PJ-DEAD, PJ-INPP5E or PJ with LYN11-FRB. B. Mean CD of currents recorded from untransfected HEK-IK1 cells (control; n = 11) and HEK-IK1 cells transiently expressing PJ (n = 9), PJ-INPP5E (n = 11) or PJ-DEAD (n = 9) with LYN11-FRB. C. Representative traces of currents recorded from HEK-IKr cells (control), and HEK-IKr cells transiently expressing PJ-DEAD or PJ with LYN11-FRB. D. Mean PTCD of currents recorded from HEK-IKr cells (control; n = 9) and HEK-IKr cells transiently expressing PJ (n = 10) or PJ-DEAD (n = 10) with LYN11-FRB. Data are presented as mean ± S.E.M. (TIF) [file pone.0186293.s001.tif]

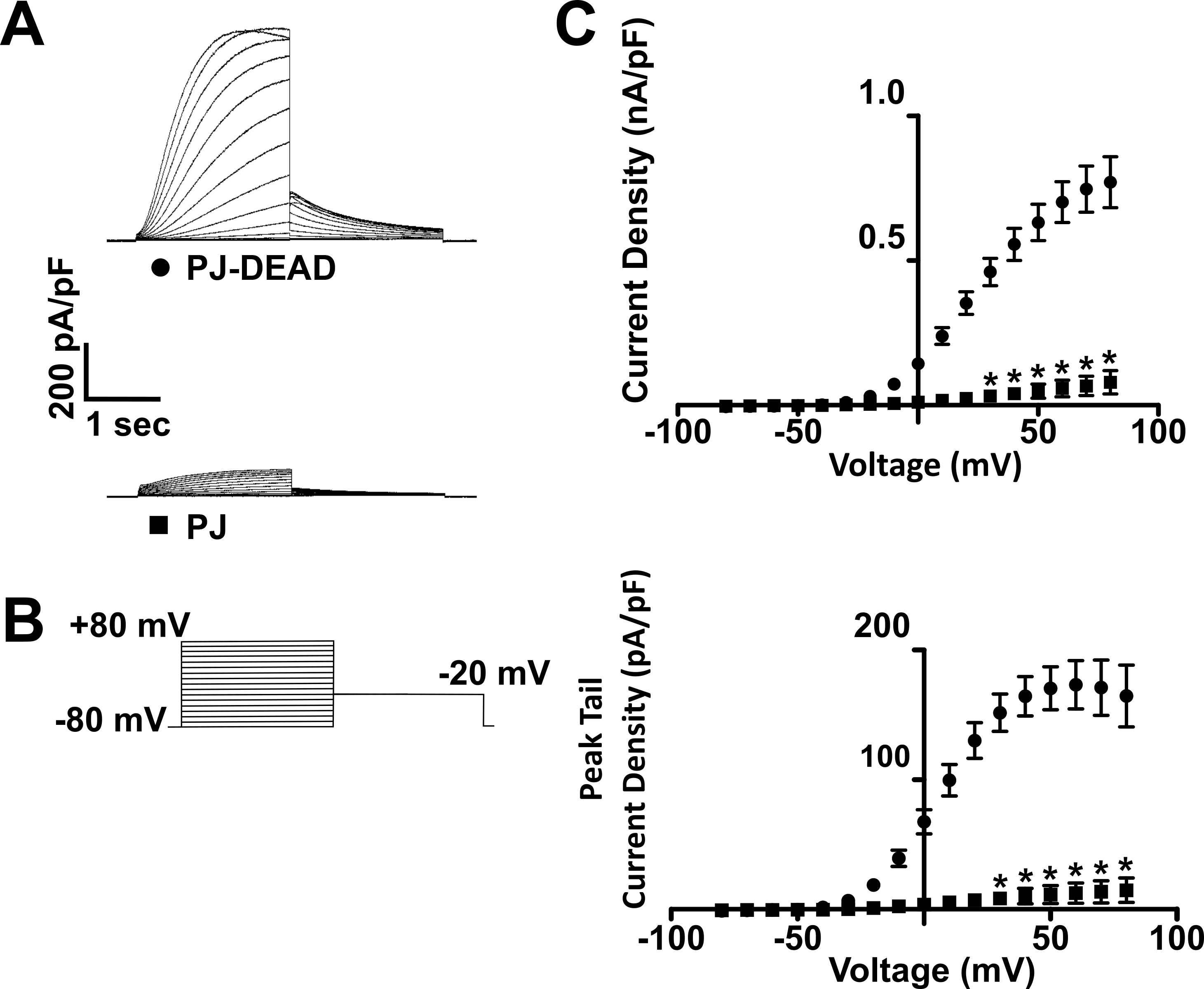

Supplement: S2 Fig — A. Representative traces of currents recorded from CHO-K1 cells transiently expressing the KCNQ1 and KCNE1 subunits with LYN11-FRB and either PJ-DEAD or PJ. B. Voltage protocol used to elicit current recorded. C. Mean CD (top) and PTCD (bottom) of currents from CHO-K1 cells transiently expressing KCNQ1 and KCNE1 with LYN11-FRB and either PJ-DEAD (n = 12) or PJ (n = 11). Data are presented as mean ± S.E.M. An unpaired t-test was performed to determine statistical significance between groups in C, at voltages between +30 mV and +80 mV. * indicates significant difference (P <0.05) from control (KCNQ1 + KCNE1 + PJ-DEAD) value. (TIF) [file pone.0186293.s002.tif]

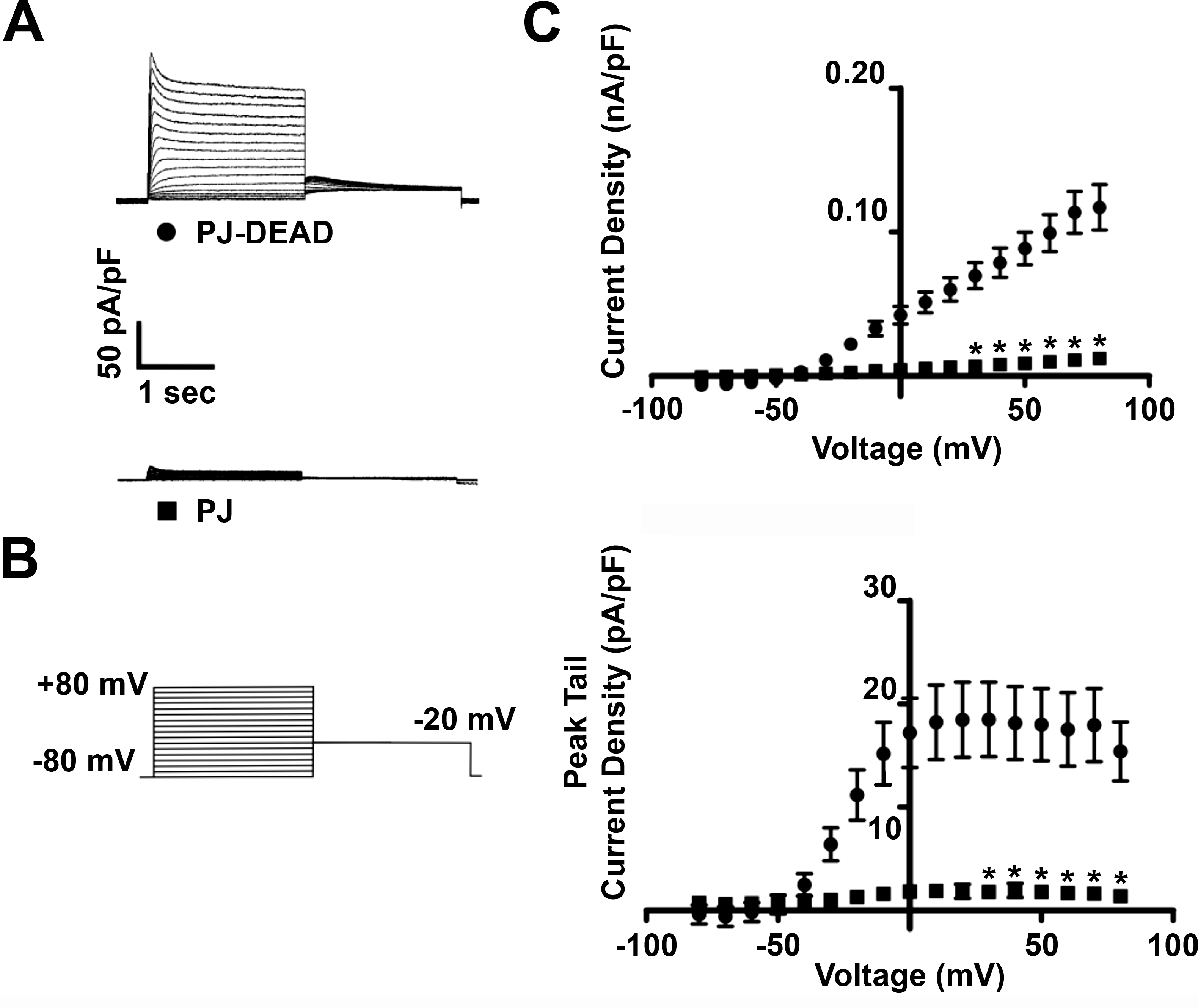

Supplement: S3 Fig — A. Representative traces of currents recorded from CHO-K1 cells transiently expressing KCNQ1 with LYN11-FRB and either PJ-DEAD or PJ. B. Voltage protocol used to elicit current recorded. C. Mean CD (top) and PTCD (bottom) of currents from CHO-K1 cells transiently expressing KCNQ1 with LYN11-FRB and either PJ-DEAD (n = 11) or PJ (n = 10). Data are presented as mean ± S.E.M. An unpaired t-test was performed to determine statistical significance between groups in C, at voltages between +30 mV and +80 mV. * indicates significant difference (P <0.05) from control (KCNQ1 + PJ-DEAD) value. (TIF) [file pone.0186293.s003.tif]

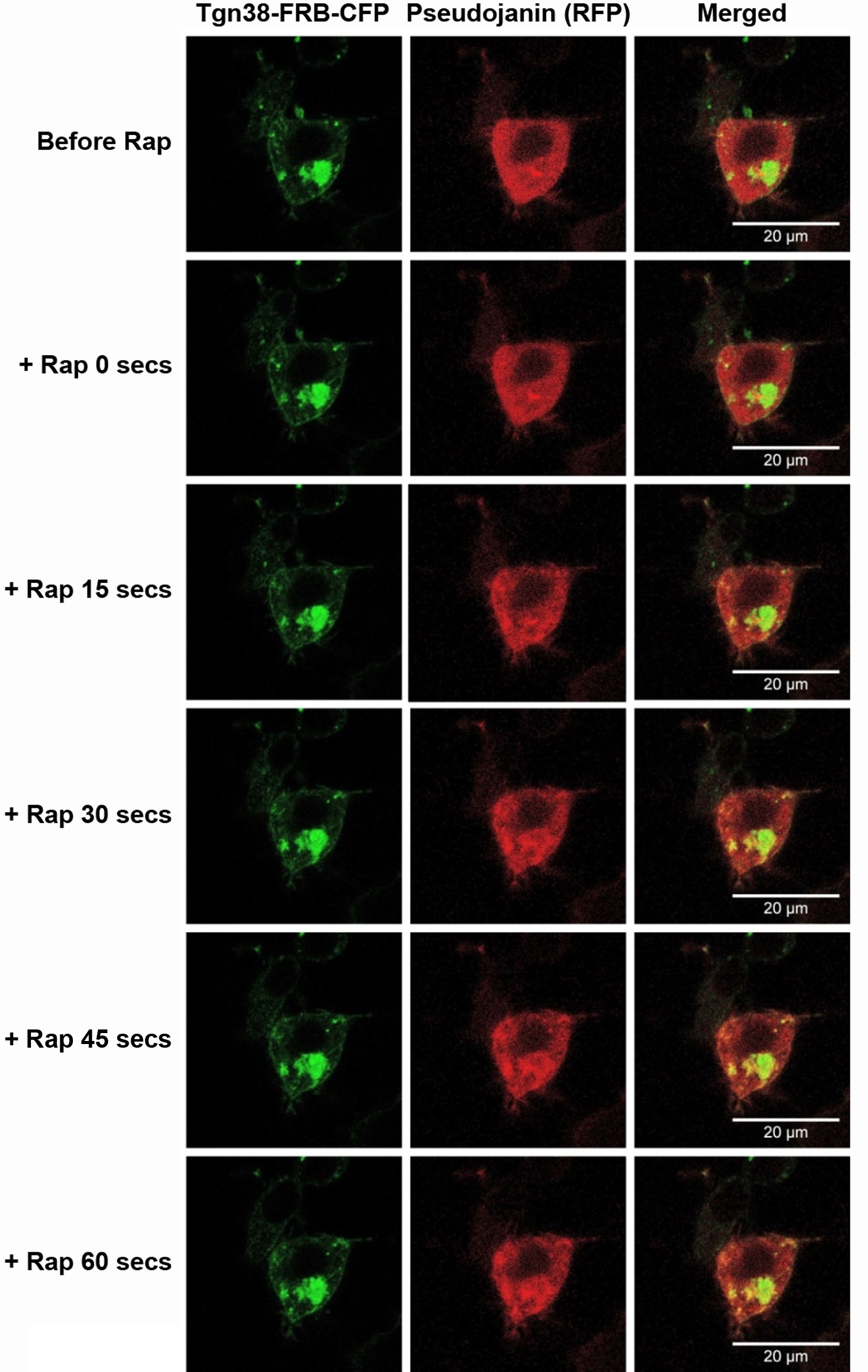

Supplement: S4 Fig — Top panel: The localisation of PJ and Tgn38-FRB in HEK293 cells. The five centre and bottom rows show the same cell at different time points after the addition of rapamycin (5 μM). Scale bar indicates 20 μm. (TIF) [file pone.0186293.s004.tif]
